# *Wolffia globosa* as an Emerging Plant-Based Protein Source for Functional and Nutraceuticals

**DOI:** 10.3390/foods15030543

**Published:** 2026-02-03

**Authors:** Karthikeyan Venkatachalam, Suphat Phongthai, Ratchanee Puttha, Jittimon Wongsa, Narin Charoenphun

**Affiliations:** 1 Faculty of Innovative Agriculture, Fisheries and Food, Prince of Songkla University, Surat Thani Campus, Makham Tia, Mueang, Surat Thani 84000, Thailand; karthikeyan.v@psu.ac.th; 2 School of Agro-Industry, Faculty of Agro-Industry, Chiang Mai University, Chiang Mai 50100, Thailand; suphat.phongthai@cmu.ac.th; 3 Faculty of Agricultural Production, Maejo University, Chiang Mai 50290, Thailand; ratchanee_pt@mju.ac.th; 4 Faculty of Industrial Technology and Management, King Mongkut’s University of Technology North Bangkok (Prachinburi Campus), Prachinburi 25230, Thailand; jittimon.w@itm.kmutnb.ac.th; 5 Faculty of Science and Arts, Burapha University Chanthaburi Campus, Chanthaburi 22170, Thailand

**Keywords:** *W. globosa*, green caviar, plant-based protein, functional foods, nutraceuticals, sustainable food, aquatic plant, bioactive compounds

## Abstract

*Wolffia globosa* (*W. globosa*), an edible aquatic plant of the Lemnaceae family, has gained increasing attention as a potential alternative protein and functional food ingredient due to its rapid biomass production, favorable amino acid profile, and micronutrient content. This review critically evaluates the current evidence on the nutritional composition, protein quality, reported bioactive properties, safety considerations, and regulatory status of *W. globosa*, focusing on its suitability for food applications. Literature data indicate that *W. globosa* biomass can contain substantial protein levels on a dry-weight basis, with reported protein quality metrics approaching those of some conventional plant proteins under specific processing conditions. In addition, studies have explored the high antioxidant, antihypertensive, and metabolism-related bioactivities of *W. globosa*, primarily based on in vitro and animal studies. However, human clinical evidence remains limited, and reported functional effects should be interpreted with caution. Regulatory assessments, including novel food authorization in certain jurisdictions, support its use as a food ingredient under defined conditions but do not substantiate health claims. Overall, *W. globosa* represents a promising plant-based food resource; nevertheless, further standardized compositional analyses, bioavailability studies, and well-designed human trials are required to substantiate its functional and nutritional properties.

## 1. Introduction

Plant-based protein innovation has expanded beyond conventional terrestrial crops as global food systems face pressures related to population growth, climate variability, land and water constraints, and the need to improve dietary quality [1,2]. Within this landscape, small aquatic plants in the Lemnaceae family have been investigated as candidate biomass resources, as they can achieve high areal productivity under controlled conditions and can be cultivated without direct competition for arable land [3,4]. *W. globosa*, a rootless, free-floating species within this family, is of particular interest for food applications because it can be produced as an edible biomass that contains substantial protein on a dry-weight basis, along with other nutrients that may be relevant for diet diversification [5]. Recently, *W. globosa* has gained global interest as a food ingredient and potential replacement for three primary reasons: (i) its rich amino acid profile and high protein yield relative to its cultivation footprint [5]; (ii) the bioactive properties of protein hydrolysates derived from *W. globosa* extracts, including antioxidant capacity and peptide-mediated effects demonstrated in model systems [6,7]; and (iii) the emergence of commercial markets in several regions [8]. These attributes position *W. globosa* as a candidate ingredient for product development, provided that composition, safety, and evidence for function are evaluated in a manner aligned with intended food-use conditions. Unlike broader reviews that discuss Lemnaceae/duckweed in general, this review focuses specifically on *W. globosa* as an edible protein ingredient and critically links composition, processing, safety, and model-specific evidence for functional effects to support realistic food and nutraceutical development.

Simultaneously, the current literature contains recurring issues that complicate the translation of these findings into food and nutrition practices. Composition and protein quality metrics are not always comparable across studies because of differences in the biomass form (fresh versus dried, powder, or isolate), analytical methods, and reporting units [9]. Bioactivity findings frequently rely on in vitro assays and animal models, which are insufficient to infer human efficacy without appropriate consideration of exposure levels, bioavailability, and well-designed trials [1,6,7,10]. In addition, regulatory decisions that permit the use of food ingredients under defined conditions should not be interpreted as substantiating health claims [10]. Safety considerations are also central, because Wolffia species can accumulate contaminants from growth water; therefore, cultivation control, sourcing, and contaminant monitoring are essential when positioning *W. globosa* for food and nutraceutical applications. These constraints highlight the need for a critical, model-stratified review to avoid overinterpretation and identify research priorities that are directly relevant to product development and responsible communication [9].

Accordingly, this review synthesizes and critically evaluates evidence on *W. globosa* with a focus on food-relevant dimensions, including taxonomic clarity and production factors that influence ingredient consistency [5]; nutritional composition and protein quality assessment, including the interpretability of amino acid and digestibility metrics [9]; reported bioactive compounds and functional properties with explicit separation of evidence by experimental model [7]; processing and application considerations that can affect nutrient stability and sensory acceptability [11]; safety risks and mitigation strategies, including contaminant control in aquatic cultivation systems; regulatory status and its practical implications for food use; and sustainability claims evaluated in the context of available life cycle evidence [10]. By linking composition, processing, safety, and evidence strength, this review aims to clarify the practical application potential of *W. globosa* for food and nutraceutical applications and to define priority research needs for standardized compositional specifications, bioavailability assessment, and human substantiation of functional outcomes.

## 2. Literature Search Strategy

This structured narrative review synthesizes and critically evaluates the published evidence on *W. globosa* as a food and nutritional resource for humans. A literature search was performed using the Scopus, Web of Science, and PubMed databases, covering publications from 2000 to 2024. An additional targeted search using Google Scholar was used to identify relevant regulatory and consensus documents and to retrieve articles not indexed in the primary databases. Search terms included combinations of “*Wolffia globosa*”, “*W. globosa*”, “duckweed protein”, “watermeal”, “Lemnaceae nutrition”, “protein quality”, “bioactive peptides”, “vitamin B12”, “functional food”, and “novel food” using Boolean operators (AND/OR) and truncation where applicable. Peer-reviewed research articles, reviews, and regulatory reports written in English were included in this study. Studies were included if they reported data on (i) nutritional composition, (ii) protein quality indices (e.g., amino acid composition, protein digestibility-corrected amino acid score (PDCAAS), or related metrics), (iii) bioactive properties (in vitro, animal, or human), (iv) safety or toxicological evaluation (e.g., contaminants, heavy metals, antinutritional compounds), or (v) regulatory assessment of *W. globosa* for food use. To ensure food relevance, only studies with clearly described edible biomass (fresh or dried biomass, powders, concentrations, isolates, hydrolysates, or food products containing *W. globosa*) were prioritized. Studies focusing exclusively on non-food applications were excluded. Specifically, studies were excluded if they (i) addressed phytoremediation or wastewater treatment without an edible-use context, (ii) lacked sufficient methodological detail to interpret compositional or functional outcomes (e.g., unclear sample identity or analytical basis), (iii) did not clearly attribute outcomes to Wolffia species, or (iv) reported outcomes not relevant to food, nutrition, safety, or functional properties. Evidence was qualitatively categorized according to the experimental model (in vitro, animal, or human) to facilitate critical comparison of evidence strength. No quantitative meta-analysis was conducted because of the heterogeneity in study designs, processing conditions, and outcome measures. Where multiple studies addressed similar endpoints, results were interpreted in relation to sample form, cultivation conditions (when available), processing steps, and analytical reporting units to improve comparability.

## 3. Botanical Description and Cultivation

### 3.1. Taxonomy and Morphological Characteristics

**Figure 1:**
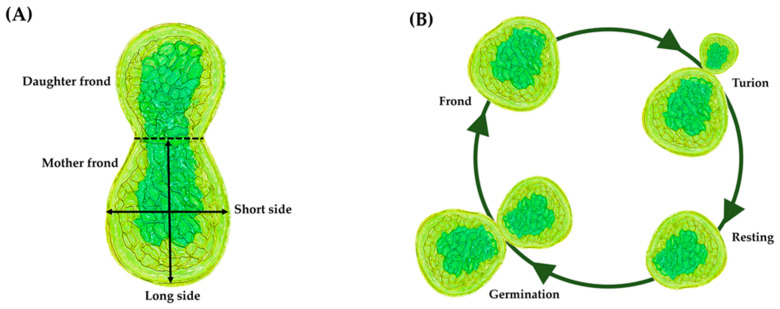
Morphology (**A**) and life cycle of *W. globosa* (**B**). Adapted from Romano et al. [15] Copyright 2024 Scientific Reports and Ziegler et al. [17] Copyright 2025 MDPI.

Taxonomically, Wolffia (commonly known as watermeal or duckweed) is classified within the Kingdom Plantae, clade Angiosperms, clade Monocots, order Alismatales, and family Araceae-a family that formerly encompassed the standalone family Lemnaceae [3,12]. The Lemnaceae family comprises five genera: Spirodela, Landoltia, Lemna, Wolffiella, and *Wolffia*. The genus Wolffia includes 11 recognized species, each characterized by highly reduced morphology, rapid asexual reproduction, and a broad geographical distribution. The species currently accepted within the genus are: (1) *W. globosa*, (2) *W. arrhiza*, (3) *W. australiana*, (4) *W. borealis*, (5) *W. brasiliensis*, (6) *W. cylindracea*, (7) *W. neglecta*, (8) *W. angusta*, (9) *W. Columbiana*, (10) *W. elongata*, and (11) *W. microscopica* [13]. Wolffia exhibits highly reduced morphological structures with no stems, roots, or true leaves. Its body is a leaf-like thallus or frond. Turions are dormant fronds that are smaller and morphologically distinct from the parent fronds. They are characterized by thicker cell walls, greatly reduced air spaces and vacuoles, and substantially higher starch accumulation than the normal fronds from which they originate [14]. The flowers are extremely small and rarely observed, emerging from a small cavity on the upper surface of the thallus. Wolffia also produces the world’s smallest fruits, which are indehiscent, bladder-like, one-seeded structures commonly referred to as utricles. Each balloon-like utricle contains a single smooth seed approximately 0.5 mm in size [3,15] (Figure 1A).

### 3.2. Growth Physiology and Reproductive Characteristics

Wolffia growth physiology is characterized by rapid cell division and efficient nutrient assimilation, positioning it as one of the fastest-growing angiosperms [16]. Vegetative budding is the primary reproductive mode, in which a mother frond generates daughter fronds within specialized pouches. Fronds largely consist of spongy mesophyll tissue containing large air spaces that make them buoyant [17]. The simplified morphology under adverse growth conditions, known as turions or winter/overwintering buds, are vegetative, dormant organs formed by Wolffia [15,18]. Figure 1B shows the life cycle of *W. globosa*.

### 3.3. Cultivation and Production Systems

Wolffia, commonly harvested from natural freshwater bodies with minimal water flow, is traditionally collected directly from ponds for culinary use. However, the consumption of wild-harvested Wolffia raises concerns regarding hygiene and potential contamination by pathogenic microorganisms naturally present in such environments, which may lead to gastrointestinal illness. Moreover, with the increasing demand for Wolffia as a nutritious food source, reliance solely on natural habitats has become insufficient to ensure a stable supply. Wild collection also limits the ability to control the yield, safety, and nutritional composition, which are critical for food quality assurance. Consequently, various cultivation and production systems have been developed to support the scalable and controlled production of Wolffia spp. (Table 1). These systems differ in terms of their structural design, operational efficiency, advantages, and their limitations. Fertilizer management plays a key role in determining the growth, biomass yield, and bioactive composition of watermeal (*W. globosa*). This simple cultivation system is suitable for producing food for both humans and animals. Watermeal was grown in outdoor polyethylene (PE) tanks with regular application of NPK (15:15:15) fertilizer over a 35-day period. This cultivation approach resulted in the highest biomass production, with a protein content of 40.64 ± 2.13% and a broad amino acid profile consisting of 17 amino acids, including nine essential amino acids (EAAs) and eight non-essential amino acids (NEAAs). Glutamic acid is the most abundant amino acid, and leucine and lysine are the predominant essential amino acids [5]. Furthermore, a small-scale experimental prototype of a recirculating aquatic indoor vertical farm (IVF) demonstrated the feasibility of applying such a system as a plant factory under artificial lighting. This approach could support the upscaled cultivation of Wolffia in commercial recirculating setups for biomass production, while highlighting the need to carefully manage potential nutrient imbalances in the stock solutions [19]. Bioreactors are specialized devices or systems designed to provide a controlled environment that supports the growth of living organisms, such as plant cells, to facilitate the production of bioactive compounds. Duckweed, due to its small size and rapid growth rate, can generate high biomass within a limited space and is easy to process, making it a promising candidate for the production of bioactive phytochemicals for pharmaceutical applications. The application of plant-based bioreactors offers several advantages, including product safety, low production costs, and scalability, which have contributed to the increasing adoption of this approach in the cultivation of duckweed. Nevertheless, bioreactor systems face certain limitations, including high operational costs, susceptibility to contamination, and cell sensitivity to changes in environmental conditions [20,21].

### 3.4. Environmental Tolerance and Ecological Benefits

Wolffia exhibits a high growth rate and strong stress tolerance, along with a remarkable capacity to accumulate substantial amounts of pollutants, making it an excellent candidate for the phytoremediation of heavy metals. According to a report by Xie et al. [23], *W. globosa* exhibits a high cadmium (Cd) accumulation capacity when exposed to low Cd concentrations, together with moderate tolerance (half-maximal effective concentration (EC_50_) for biomass of 4.80 μM), indicating its strong potential for environmental remediation. Chromium (Cr) is classified as a heavy metal that is carcinogenic to humans. The WHO recommends that Cr levels in drinking water should not exceed 0.05 mg/L, and Cd levels should be within 0.003 mg/L. Research has shown that *W. globosa* can effectively accumulate Cd, whereas its Cr-accumulating capacity is moderate. The plant can tolerate both Cd and Cr, particularly at low to moderate concentrations (1–4 mg/L). Therefore, *W. globosa* may be a useful species for the remediation of aquatic environments contaminated with heavy metals [24]. Another species within the genus Wolffia that has been frequently cited for its notable efficiency in heavy metal uptake and accumulation is *Wolffia arrhizal* (*W. arrhizal*). *W. arrhiza* cultivated in catfish pond wastewater with varying contaminant levels over a 7-day period exhibited notable treatment efficiency. Optimal performance was observed at an initial organic loading of 0.1 g chemical oxygen demand (COD) per gram of plant, resulting in the removal of 15% COD, 80% biological oxygen demand (BOD), 99% ammoniacal nitrogen (AN), and 94% total suspended solids (TSS). Furthermore, *W. arrhiza* obtained following the phytoremediation process demonstrated promising suitability as a fish feed ingredient, provided that its nutritional formulation is appropriately enhanced [25]. Assessing the impact of pollutants in municipal and industrial wastewater on aquatic ecosystems, including aquatic plants, is essential for planning and improving phytoremediation strategies. In this context, the use of *W. arrhiza* to examine low-level contamination by bisphenol A (BPA), N, N-diethyl-m-toluamide (DEET), triclosan (TRC), benzophenone (BPH), and endosulfan isomers (α-END and β-END) provides valuable insights into the biological toxicity of organic micropollutants (OMPs) and their effects on plant growth [26]. However, harvesting Wolffia from potentially contaminated water bodies, particularly for food and feed purposes, should be carried out with caution and under strict monitoring because of its high capacity to accumulate pollutants.

### 3.5. Challenges in Cultivation

In the present and future food sectors, there is an increasing demand for plant-derived ingredients that offer health benefits and can be produced under high safety standards with minimal chemical contaminants. Wolffia has emerged as a promising alternative crop due to its high protein content, complete essential and non-essential amino acid profile [27], pigments, and bioactive compounds, making it suitable as a future food source [28]. Traditionally, Wolffia has been collected from natural water bodies and consumed at the household level without the use of advanced production technologies. This reliance on wild harvesting has resulted in technological gaps, particularly in controlled cultivation, product consistency, and food safety. Therefore, greater support from the government and private sectors in producing countries is essential to promote research, standardize production systems, and scale-up Wolffia for safe and reliable food applications. The rising consumer demand has stimulated rapid progress in cultivation technologies for space agriculture [29]. However, research on breeding and genetic improvement and the lack of reliable methods for stable genetic transformation [30] to obtain high-yielding, safe, and nutritionally consistent cultivars remain limited and require further investigation.

## 4. Nutritional and Phytochemical Composition

### 4.1. Proximate Composition

*W. globosa* is characterized by a nutrient-dense macronutrient profile in its dried form, with comparatively high carbohydrate and protein fractions and substantial fiber content, supporting its potential as a food ingredient (Table 2). Seephua et al. [7] reported the proximate composition of *W. globosa* powder (g/100 g dry weight, DW) with carbohydrates of 50.73–52.44, protein of 20.55–22.74, fiber of 15.76–16.53, ash of 6.76–7.84, moisture of 3.73–4.25, and fat of 3.23–4.08. The authors noted that these values were influenced by the growing environment, which is consistent with the observed variability commonly reported for aquatic biomass cultivated under different nutrient and water quality conditions. Comparable findings were reported by On-Nom et al. [27], who found the composition of *W. globosa* powder (g/100 g DW) to be 52.59 g carbohydrates, 36.52 g dietary fiber, 31.50 g protein, 10.73 g ash, and 5.18 g fat. Collectively, these reports indicate that dried *W. globosa* can provide a carbohydrate-dominant macronutrient base while maintaining meaningful protein content and a high fiber fraction, although the magnitude of fiber and protein can differ substantially between studies, emphasizing the need to report the cultivation and processing context when comparing the compositions across sources. On an energy basis, dried *W. globosa* has been reported to provide approximately 200–383.03 kcal per 100 g DW.

### 4.2. Amino Acid Profile and Protein Quality

Essential amino acids in *W. globosa*, such as arginine (1.32–2.13), phenylalanine (1.01–1.36), valine (1.03–1.67), isoleucine (0.92–1.32), and leucine (0.87–1.24), occur in proportions exceeding those of tryptophan (0.45–0.58), lysine (0.29–0.47), histidine (0.24–0.42), methionine (0.14–0.18), and threonine (0.12–0.22) [7]. The amino acids found in *W. globosa* are similar to those found in the proteins of legumes. The protein digestibility-corrected amino acid score (PDCAAS) of *W. globosa* is 89% (or 0.89), indicating excellent protein quality for a plant protein source [31]. The PDCAAS of plant-based proteins was reported by Anyiam et al. [9] for Bambara nut (0.32–0.68), moringa seed (0.41–0.68), rice bran (0.63–0.90), mung bean (0.43–0.68), jack bean (0.62–0.70), and soybean (0.92–1.00). Furthermore, enzymatic hydrolysis modified the secondary structure of *W. globosa* proteins by converting beta sheets and random coils into alpha-helices and beta turns. Hydrolysis increased protein solubility and emulsifying activity, and partial hydrolysis of *W. globosa* enhanced foaming and emulsifying properties [1,27,32,33,34]. Bioengineered peptides have demonstrated potential inhibitory effects on human ovarian cancer cells [1], antioxidant [32], antimicrobial [33], and antihypertensive properties [6,34].

### 4.3. Lipid Composition

Palmitic acid was the most abundant saturated fatty acid in *W. globosa* (24.5–25.5% of the fatty acid composition). Stearic, lauric, myristic, and capric acids were found in varying amounts depending on the breeding ground. Furthermore, high levels of polyunsaturated fatty acids (PUFAs), particularly linoleic and alpha-linolenic acids, were present. Approximately 30% of the total fatty acids are α-linolenic acid (C18:3 ω3), which is the most abundant PUFA [7]. The nutritional potential of *W. globosa* as a plant-based source of omega-3 fatty acids is indicated by its high α-linolenic acid content. The control of inflammation, brain development, cognition, and retinal health all depends on these fatty acids. Campesterol, cycloartenol, stigmasterol, brassica sterol, and β-sitosterol have also been identified in *W. globose* [6,7]. Phytosterols are natural plant substances that belong to a subgroup of steroid alcohols with a chemical structure similar to that of cholesterol, which is found in animals. However, they are beneficial in reducing the absorption of low-density lipoprotein (LDL) cholesterol into the body, thereby lowering blood cholesterol. Furthermore, β-sitosterol possesses antioxidant, antimicrobial, anti-arthritic, and pain-reducing properties without significant toxicity [6].

### 4.4. Carbohydrates and Dietary Fiber

*W. globosa* contains carbohydrates as its primary constituent, which vary greatly depending on the species, location, and growing conditions. Various beneficial carbohydrates, including cellulose, pectin, and hemicellulose, are considered dietary fibers. *W. globosa*’s carbohydrate and dietary fiber content is approximately 38.41%, total starch is 10.39%, total dietary fiber is 20.65%, insoluble dietary fiber is 18.37%, and soluble dietary fiber is 2.29% [35]. *W. globosa* has been shown to affect postprandial glycemic response. It may have beneficial effects on blood sugar control and is a good dietary option for those at risk of or with diabetes [36]. Moreover, soluble dietary fibers help manage blood sugar and lipid levels by binding to water and forming a gel that helps the digestive system function better [37].

### 4.5. Micronutrients

*W. globosa* can produce vitamin B12 by accommodating duckweed-associated bacteria, which live in the environment of plants in the Lemnaceae family. It could be an interesting source of vitamin B12 in plant foods, especially as a substitute for vitamin B12 in vegetarian or plant-based diets that may lack this vitamin [38]. Appenroth et al. [39] reported that the micronutrients of plants in the Lemnaceae family are rich in β-carotene and α-tocopherol. Mineral analysis revealed the presence of calcium (Ca), potassium (K), magnesium (Mg), sodium (Na), phosphorus (P), iron (Fe), manganese (Mn), copper (Cu), zinc (Zn), iodine (I), and selenium (Se) in *W. globosa*.

### 4.6. Phytochemical Composition and Bioactive Compounds

Phytochemical analysis has identified diverse phenolic acids (gallic, protocatechuic, vanillic, caffeic, syringic, p-coumaric, ferulic, sinapic, cinamic, and genistic acids) and flavonoids (rutin, quercetin, apigenin, and karmferal) in *W. globosa* extracts [7]. It also contains high amounts of chlorophyll, carotenoids and flavonoids [40]. These compounds possess powerful antioxidant properties and enhance the nutritional value of foods [32]. Phenolic metabolomics of *W. globosa* revealed approximately 200 polyphenols and phenolic metabolites with high phenolic and antioxidant contents, with a high concentration of flavonoid polyphenols, such as luteolin and apigenin derivatives [7,32,41]. The health benefits of flavonoids, due to their biological activities, include antioxidant, hepatoprotective, and anti-inflammatory properties, and some studies have suggested their anticancer activities [41]. Phytochemical composition and bioactive compounds vary depending on environmental conditions, nutrient availability, and post-harvest processing, highlighting the need for standardization of the composition of commercial products [38].

### 4.7. Comparative Nutritional Perspective

*W. globosa* exhibits a distinctive and highly competitive nutritional profile compared to conventional and emerging plant-based protein sources, including soybean, pea, and spirulina (*Limnospira platensis*) (Table 3). Although its protein content (20–30% dry weight) is lower than that of soybean and spirulina, *W. globosa* achieves an exceptionally high protein yield per hectare, approximately three times greater than that of soybean, which underscores its superior land-use efficiency [16]. In addition to protein quantity, the amino acid composition of *W. globosa* provides substantial levels of essential amino acids, fulfilling human nutritional requirements, especially when incorporated into diverse diets [27]. *W. globosa* also delivers notable nutritional advantages through its favorable lipid profile, which includes a high proportion of polyunsaturated fatty acids and an n-6/n-3 ratio consistently below 1.0, a value rarely observed in terrestrial plant proteins [16]. Unlike microalgae such as Spirulina, *W. globosa* does not contain cyanotoxins, thereby reducing food safety risks and streamlining regulatory and processing considerations for food applications [29]. The abundance of micronutrients and bioactive compounds, including vitamin E (α-tocopherol), lutein, iron, dietary fiber, and potent antioxidants such as γ-oryzanol, positions *W. globosa* as a nutrient-dense functional ingredient with significant potential to improve the nutritional quality and sustainability of future food systems [16].

## 5. Bioactive and Functional Properties

### 5.1. Antioxidant and Anti-Inflammatory Activities

Wolffia species are a concentrated source of antioxidant and anti-inflammatory bioactives with potential relevance to functional food applications targeting oxidative stress-related disorders. In vitro assays consistently demonstrate the strong radical-scavenging capacity of *W. globosa* and *W. arrhiza* extracts (DPPH, ABTS, and FRAP), which correlates positively with total phenolic and flavonoid contents, indicating a polyphenol-driven mechanism [32,43,44]. Chemical profiling has revealed a diverse antioxidant system comprising phenolic acids (e.g., protocatechuic, vanillic, and p-coumaric acids), carotenoids, vitamin C, and lipid-soluble antioxidants such as α-tocopherol and γ-oryzanol [7,43]. In parallel, protein-derived components, including vicilin-like proteins and protein hydrolysates, contribute to redox modulation, suggesting both enzymatic and non-enzymatic antioxidant pathways [33,44]. Targeted cultivation strategies further enhance bioactivity, as evidenced by selenium-enriched Wolffia, which produces specialized metabolites (e.g., indole-3-acryloylglycine and spiculosine) with enhanced antioxidant effects [45].

Anti-inflammatory activity is mechanistically linked to the suppression of proinflammatory mediators. Wolffia protein and phenolic extracts significantly reduced IL-1β and IL-6 secretion in THP-1 monocytic cells, indicating the modulation of cytokine-driven inflammatory signaling [46]. Additionally, phytosterols such as β-sitosterol and stigmasterol inhibit nitric oxide production in activated macrophages, suggesting interference with inducible inflammatory pathways [6]. Moreover, *W. globosa* is rich in essential amino acids, omega-3 fatty acids, polyphenols, iron, vitamin B12, folate, and beta-carotene, all of which play crucial roles in brain function and memory [39,40,47]. *W. globosa* may have a protective effect against neurodegeneration and could help prevent or slow the progression of diseases such as Alzheimer’s disease and age-related cognitive decline through antioxidant and anti-inflammatory mechanisms. *W. globosa* can help reduce oxidative stress, a major cause of nerve cell degeneration in Alzheimer’s and other neurological disorders. Flavonoids and phenolic compounds in *W. globosa* may also reduce inflammation by decreasing the release of pro-inflammatory cytokines associated with brain swelling [9,41,48].

**Figure 2:**
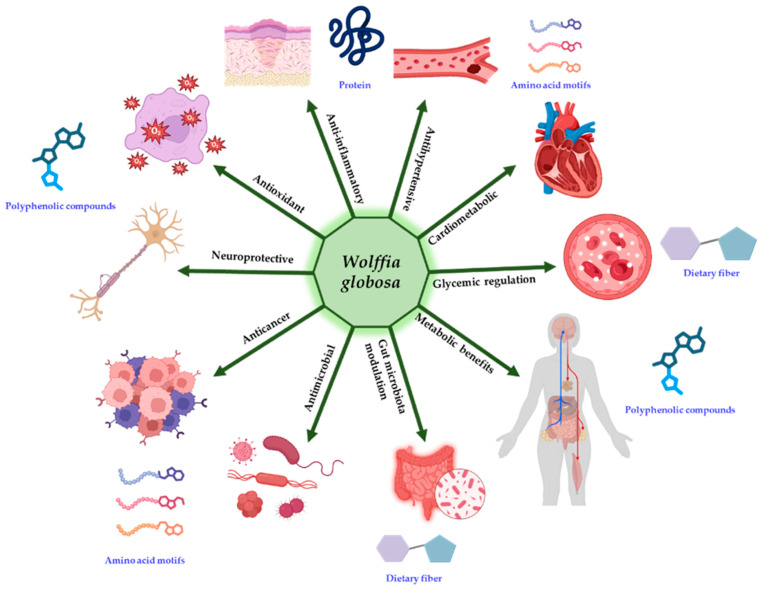
Conceptual overview of the bioactive and functional properties of *W. globosa* and its major constituent classes.

Although these findings strongly support redox regulation and cytokine suppression as the primary mechanisms, direct in vivo validation of canonical signaling pathways, including Nrf2–ARE activation and NF-κB inhibition, remains limited. Nevertheless, the convergence of antioxidant and anti-inflammatory actions provides a mechanistic foundation for the reported cardiometabolic benefits of Wolffia as a functional food ingredient (Figure 2).

### 5.2. Antihypertensive and Cardiometabolic Effects

The cardiometabolic benefits of Wolffia are mechanistically linked to the redox–inflammation axis described in Section 5.1, with direct implications for blood pressure and lipid regulation. Protein hydrolysates from *W. globosa* exhibit significant angiotensin-converting enzyme (ACE) inhibitory activity, attenuating angiotensin II formation and vascular constriction [33]. This effect is primarily associated with short-chain peptides enriched in hydrophobic amino acid motifs (e.g., Val–Pro–Pro and Ile–Pro–Pro), which are functionally comparable to established antihypertensive peptides from animal sources but are derived entirely from plants. In parallel, Wolffia consumption improves lipid metabolism in animal models, as reflected by reductions in serum low-density lipoprotein cholesterol and triglyceride levels [33]. These effects are mediated through complementary mechanisms, including enhanced bile acid excretion by soluble dietary fiber and suppression of lipid peroxidation by phenolic antioxidants, thereby reinforcing the oxidative stress–inflammation–cardiometabolic continuum [9,41,45,48]. Collectively, the convergence of ACE inhibition, lipid modulation, and antioxidant–anti-inflammatory actions support *W. globosa* as a multifunctional ingredient for cardiometabolic health and the prevention of metabolic syndrome [33].

### 5.3. Glycemic Regulation and Metabolic Benefits

Wolffia demonstrates consistent glycemic regulatory effects, supporting its potential role in dietary strategies for targeting metabolic syndrome and type 2 diabetes. Wolffia-based foods exhibit hypoglycemic activity primarily through a low glycemic impact associated with slow-digesting carbohydrates and high dietary fiber content, resulting in attenuated postprandial glucose responses [27]. Human clinical trials in Thailand further confirmed reduced postprandial glycemia and increased satiety following the consumption of *W. globosa*-enriched meals compared with isoenergetic control meals [27]. At the mechanistic level, glycemic modulation is linked to the combined effects of dietary fiber and polyphenolic compounds. Polyphenols in Wolffia inhibit key carbohydrate-hydrolyzing enzymes, including α-amylase and α-glucosidase, thereby slowing glucose release and its intestinal absorption. Together, fiber-mediated delayed digestion and enzyme inhibition provide a coherent mechanistic basis for the metabolic benefits of *W. globosa*, supporting its application as a functional ingredient for glycemic control and metabolic health management [27].

### 5.4. Gut Microbiota Modulation

*W. globosa* is a fiber-containing plant-based food with potential relevance to gut health, given the established role of dietary fiber in supporting microbial fermentation and metabolic activity. Soluble fiber and resistant starch from plant foods serve as fermentable substrates for saccharolytic bacteria, resulting in the production of short-chain fatty acids (SCFAs), such as acetate and butyrate, which contribute to gut barrier integrity and host metabolic regulation. However, direct evidence demonstrating the selective modulation of gut microbiota by *W. globosa* remains limited. Recent in vitro colonic fermentation studies using *W. globosa* digesta and its polysaccharide- and protein-rich extracts have reported increased growth of putatively beneficial microbial taxa (e.g., Bifidobacteriaceae, Bacteroidaceae, Megamonas, and Blautia) and elevated SCFA production compared with control substrates [49]. While these findings suggest that duckweed-derived components may act as fermentable substrates for the gut microbiota, evidence is currently restricted to in vitro models, and further in vivo and human clinical studies are required to confirm their functional effects.

### 5.5. Antimicrobial and Anticancer Potential

Wolffia extracts have demonstrated antimicrobial activity relevant to food safety and preservation, although the available evidence is primarily derived from in vitro studies conducted in laboratories. Extracts from *W. globosa*, including protein concentrate hydrolysates, protein extracts, and protein solutions, exhibit inhibitory effects against Gram-positive bacteria, such as *Staphylococcus aureus* and *Listeria monocytogenes,* as well as selected foodborne pathogens and spoilage organisms, including *Vibrio parahaemolyticus* and *Candida albicans* [33]. These effects are mainly attributed to phenolic acids and phytosterols, which impair microbial membrane integrity and suppress microbial growth. In parallel, limited in vitro studies have reported the cytotoxic or growth-inhibitory effects of Wolffia-derived compounds in selected cell culture models. Protein hydrolysate-derived amino acids and specialized metabolites from selenium-enriched Wolffia (e.g., indole-3-acryloylglycine and spiculosine) have demonstrated anti-proliferative activity in cancer cell lines; however, these findings remain exploratory and lack validation in vivo or in food-related contexts [1,45]. Consequently, current evidence supports the relevance of Wolffia primarily as a source of natural antimicrobial agents with potential applications in food preservation, while claims of anticancer activity should be interpreted with caution pending further mechanistic and translational studies.

### 5.6. Functional Properties of Wolffia Proteins in Food Systems

The applicability of *W. globosa* proteins in food systems is supported by their favorable techno-functional properties, which are comparable to those of conventional plant protein ingredients [31,44,50]. At neutral pH, Wolffia proteins exhibit high water solubility (>80%), facilitating their incorporation into liquid and emulsified formulations [51]. Emulsifying performance, with reported emulsifying activity indices of approximately 30–50 m^2^·g^−1^, indicates effective stabilization of oil–water interfaces [50,52]. In addition, their measurable foaming capacity, along with good water- and oil-holding properties, supports their application in structured and aerated food matrices [1,44]. These functionalities are primarily attributed to the compositional characteristics of Wolffia protein fractions, including protein concentrate hydrolysates, protein extracts, and soluble protein solutions, which exhibit enhanced interfacial activity and binding capacity [33]. Specific storage proteins, such as vicilin-like proteins, further contribute to water and oil retention, and heat-induced gelation expands the applicability of these proteins in thermally processed foods [44,53]. As summarized in Table 4, the identified bioactive compounds and associated functional activities highlight the dual role of *W. globosa* proteins in delivering both techno-functional performance and bioactivity, supporting their potential as versatile plant-based ingredients in clean-label food formulations.

Overall, the evidence suggests that *W. globosa* may support health-related outcomes through multiple mechanisms, including antioxidant and anti-inflammatory activity, peptide-mediated ACE inhibition, and fiber–polyphenol effects relevant to postprandial glycemia. However, the strength of evidence differs by endpoint, with many findings derived from in vitro assays or animal models. Therefore, clinical substantiation will require standardized ingredient specifications, defined intake levels in realistic food formats, and well-designed human trials that measure clinically relevant endpoints.

## 6. Functional and Nutraceutical Ingredients

### 6.1. Potential Bioactive Compounds and Prospective Development Strategies

*W. globosa* comprises various naturally occurring bioactive compounds, some of which can be readily extracted and utilized in their native forms. Phenolic acids and flavonoids are the predominant bioactive compounds in numerous edible plants, including *W. globosa* [43,45,54]. Protocatechuic acid, gallic acid, quercetin, and apigenin are routinely detected and associated with antioxidant capacity [7]. Furthermore, *W. globosa* is proposed as an abundant source of phytosterols, including β-sitosterol, stigmasterol, and vitamin B12 [6,50]. However, the composition of these compounds may vary due to numerous factors, including species, cultivation sites, light exposure, temperature, nutrient availability, and extraction conditions. In addition to the standard extraction methods for obtaining bioactive compounds for direct application, various techniques have been explored to enhance the bioactivity of compounds in *W. globosa*, minimize the impact of uncontrollable factors, and allow for increased precision in the development of high-value functional constituents. Focusing on one such technique, biotransformation in plant cells represents a potential biological process for stimulating the biosynthesis of phenolic compounds or generating novel derivatives with altered structures and functions. For example, selenium biotransformation effectively promotes the accumulation of bioactive compounds, notably indole-3-acryloylglycine and spiculosine, in *W. globosa* [45,54].

Furthermore, as *W. globosa* is widely regarded as a novel protein source, enhancing the bioactivity of its native proteins is an alternative and effective approach. The selenium biotransformation pathway has been identified as a promising biosynthetic process for producing bioactive selenoamino acids, such as selenocysteine and selenomethionine, through the absorption of selenium, which plants store via metabolic processes. The synthesis of selenoamino acids into selenoproteins has promising potential for various bioactivities, including antioxidant effects, anticancer properties, and angiotensin-converting enzyme inhibition [45,54]. Enzymatic hydrolysis is another crucial method used to cleave and release functional regions within native protein structures, leading to increased protein bioactivity. Protamex and Alcalase, widely utilized commercial enzymes, efficiently cleave peptide bonds, releasing active peptides that contribute to antioxidant and anticancer properties [1]. As each enzyme possesses a unique catalytic mechanism, the use of distinct individual enzymes or combinations of enzymes may be advantageous for producing bioactive peptides with diverse activities. However, the unhydrolyzed form of protein has also been reported to exhibit antioxidant properties and reduce pro-inflammatory cytokine production [46].

### 6.2. Bioactivities and Possible Mechanisms Associated with W. globosa Proteins

The proteins and derivatives obtained through native isolation, biotransformation, and enzymatic modification exhibit numerous bioactivities (Table 5)**.** According to Pakdeebamrung et al. [54], selenopeptides derived from Se-enriched *W. globosa* exhibit greater antioxidant activity than conventional peptides, as Se enrichment markedly enhances their free radical-scavenging capacity, leading to improved redox properties. Furthermore, selenopeptides exert an inhibitory effect on lung cancer cells (A549) through the interaction of selenium-binding protein 1 (SELENBP1) in cancer regulation, promoting apoptosis and significantly reducing the proliferation, migration, and invasive capacity of cancer cells [54]. The protein derived from *W. globosa* without hydrolysis significantly reduced the secretion of interleukin 1β (IL-1β), a key regulator of the inflammatory response, in LPS-stimulated THP-1-derived macrophages. Furthermore, the isolated protein may suppress IκB-α phosphorylation and NF-κB translocation, consequently decreasing the expression of COX-2. These dual actions endorse *W. globosa* protein as a viable candidate for anti-inflammatory nutraceuticals [46]

Plant-derived protein hydrolysates exhibit bioactivity, including anticancer activity. Bioactivity is influenced by numerous factors, including peptide size, amino acid sequence, and hydrophobicity, which are affected by the protein source and enzymes used. Bioactive hydrophobic peptides derived from *W. globosa* can chemically interact with the membrane bilayers on the outer leaflets of the human ovarian cancer cell line (A2780), thereby inducing apoptosis and inhibiting the cell cycle [1]. Furthermore, peptides may suppress certain microorganisms, such as *V. parahaemolyticus* and *C. albicans* [33]. This inhibitory mechanism is hypothesized to arise from electrostatic interactions between the positively charged peptide regions and anionic bacterial membrane surfaces, leading to increased membrane permeability and subsequent leakage of cellular content [55].

Other forms of *W. globosa* utilization have been documented for their beneficial effects. Extracts of *W. globosa*, which are abundant in β-sitosterol and stigmasterol, demonstrated anti-inflammatory activity by inhibiting nitric oxide production in RAW 264.7 macrophages [6]. Furthermore, incorporating *W. globosa* into Mediterranean diets and shakes offers beneficial effects, including elevating serum B12 levels, regulating postprandial glycemic response, maintaining iron homeostasis in humans, and effectively reversing anemia in rats [36,50,56].

**Table 5:** Bioactivities of the tested compounds/products and their possible mechanisms.

Tested Compounds/Products	Activity	Possible Mechanisms/Effects	References
Selenopeptides	Antioxidants	Improving redox properties.	[54]
	Anti-cancer (lung cancer cells; A549)	Slowing downregulation and reversing tumor progression.	[54]
Extracted protein (non-hydrolyzed)	Anti-inflammatory (reduction of IL-1β)	Downregulating phospho-NF-κB, phospho-IκB-α, and COX-2, consistent with reduced NF-κB pathway activation.	[46]
Protein hydrolysates (hydrolyzed by Protamex with DH9%)	Anti-cancer (human ovarian cancer cell line; A2780)	Bioactive hydrophobic peptides increase the interactions between anti-cancer peptides and the membrane bilayers on the outer leaflets of tumor cells, inducing apoptosis and suppressing the cell cycle.	[1]
Protein hydrolysates (hydrolyzed by Alcalase)	Anti-microbial	Inhibition of *V. parahaemolyticus* and *C. albicans* by 0.43 ± 1.31 log reduction (66.21%) and 3.70 ± 0.11 log reduction (99.98%).	[33]
*W. globosa* extracts (rich in β-sitosterol and stigmasterol)	Anti-inflammatory	Inhibition of nitric oxide production in RAW 264.7 macrophage cells.	[6]
*W. globosa*-containing Mediterranean diet	Source of vitamin B12	Increasing B12 level in serums.	[50]
*W. globosa*-containing shake	Glycemic control	Dietary fibers and polyphenols may contribute to beneficial postprandial glycemic effects.	[36]
*W. globosa*-containing Mediterranean diet (+physical activity)	Iron maintaining	Preserving iron-homeostasis in humans and efficient in reversal of anemia in rats.	[56]

### 6.3. Impact of Processing on Nutritional and Functional Quality

Limitations in product preservation and quality control for the most popular processing method of *W. globosa* include drying. However, traditional drying alters the quality of *W. globosa*, particularly its color and free radical content. It also affects consumer acceptance of the product. The effect of heat treatment during processing on the quality changes of *W. globosa* was investigated [11,57]. Optimal processing was found to preserve the green color of dried W. globosa, as assessed by chlorophyll retention and resulting color values. One popular method for preserving color, especially the green color of the raw material, is to use heat to inhibit the enzyme polyphenol peroxidase (PPO), which causes browning in fruits and vegetables, along with salt solutions to replace metal ions in the chlorophyll structure that can be lost due to heat treatment [58]. Excessive moisture content in fresh *W. globosa* is a significant obstacle to value chain development and the full utilization of its potential applications [58]. Cell lysis and dehumidification before drying fresh *W. globosa* resulted in the extraction of 560–650 mL of water per kg of fresh *W. globosa*, significantly reducing the pre-drying moisture levels and decreasing drying time and costs (Yadav et al.) [43]. The impact of processing on nutritional and functional quality is shown in Table 6.

## 7. Safety, Toxicology, and Regulatory Perspectives

### 7.1. Food Safety and Contaminant Considerations

Ensuring the safety of *W. globosa* is essential for its acceptance in the global market. As an aquatic plant, *W. globosa* can absorb nutrients and trace elements directly from water, raising concerns regarding the accumulation of heavy metals and organic contaminants when cultivated in uncontrolled environments [13]. However, studies in Thailand and Israel have shown that Wolffia grown in clean freshwater or controlled aquaponic systems contains heavy metal levels well below the maximum residue limits established by the EFSA and Codex Alimentarius [62]. Microbiological safety is a key consideration. Although Wolffia may harbor commensal microorganisms, post-harvest washing, hygienic drying, pasteurization, and freeze-drying effectively reduce microbial loads without compromising nutritional quality [11,27]. Accordingly, industrial production emphasizes Good Aquaculture Practices (GAP) and hazard analysis and critical control point (HACCP)-based control systems.

From a regulatory perspective, *W. globosa* is evaluated within the novel food framework in many regions. In the European Union, it falls under Regulation (EU) 2015/2283, which requires evidence of compositional consistency, controlled cultivation, and effective contamination management [62]. In the United States, Wolffia (Mankai^®^) has been notified as generally recognized as safe (GRAS) for use in selected food categories, supported by comprehensive safety data [63]. In countries with a history of traditional consumption, regulatory oversight primarily focuses on compliance with the national food safety standards [31].

### 7.2. Antinutritional Factors and Digestibility

Similar to many plant-based foods, *W. globosa* contains certain antinutritional compounds, including oxalates, tannins, and phytic acid, which may chelate minerals and slightly reduce the bioavailability of nutrients. Quantitative data on anti-nutritional factors in Wolffia species indicate generally low to moderate levels. For instance, *W. arrhiza* has been reported to contain oxalates at 8.04 ± 0.38 mg/g dry weight, phytic acid at 0.22 ± 0.02 mg/g dry weight, and tannins at 9.83 ± 0.55 mg/g dry weight [33]. For *W. globosa*, available data show tannin contents ranging from 17.2 ± 3.7 to 20.0 ± 1.4 mg/g dry weight [64]. Although direct measurements of oxalates and phytic acid in *W. globosa* remain limited, these antinutritional compounds are generally present at levels comparable to those found in many commonly consumed plant-based foods and can be effectively reduced through processing methods, such as blanching or fermentation [65]. In vitro digestibility assays have demonstrated high protein availability, with values up to 70.45% reported for processed samples, indicating that these factors do not significantly compromise their nutritional potential [35]. Compared with legumes, Wolffia exhibits markedly lower levels of trypsin inhibitors and lectins, making it a gentle and hypoallergenic protein source [16].

### 7.3. Allergenicity Assessment

To date, there have been no reports of allergenic responses to the consumption of *W. globosa* in humans. Comparative proteomic analyses revealed limited homology between Wolffia proteins and known allergenic proteins from soy, wheat, and nuts. Nevertheless, formal allergenicity testing through simulated gastric digestion and immunoassays is recommended for regulatory submissions to ensure consumer safety, particularly in populations with multiple food allergies [62].

### 7.4. Toxicological Studies

Animal studies have supported the safety of *W. globosa*. Subchronic oral toxicity studies in rats (up to 2 g·kg^−1^ body weight per day for 90 days) reported no adverse effects on growth, hematological parameters, or organ histopathology [66]. Genotoxicity assays, including the Ames and micronucleus tests, showed no mutagenic potential [66]. Human pilot studies involving the daily consumption of Wolffia-based products for up to 12 weeks reported no gastrointestinal or biochemical abnormalities [31]. These findings are consistent with the EFSA and FDA toxicological requirements for novel plant-based foods, which integrate repeated-dose toxicity, genotoxicity, and human tolerance data [62]. In addition, no allergenicity concerns have been identified, as Wolffia proteins show low homology to known allergens and are readily digested in simulated gastrointestinal (GI) models. Overall, the available toxicological evidence supports the safe use of *W. globosa* as a food ingredient under the intended conditions of use, although continued post-market monitoring is recommended.

### 7.5. Regulatory Status and Novel Food Approval

In 2022, the European Food Safety Authority (EFSA) granted *W. globosa* novel food approval (Commission Implementing Regulation (EU) 2022/2223), allowing its use in smoothies, soups, and functional products in the European Union [63]. The approval followed a comprehensive evaluation of the composition, toxicology, and nutritional equivalence of the data submitted by manufacturers [62]. The U.S. Food and Drug Administration (FDA) has classified Wolffia ingredients as GRAS for specific formulations [63], whereas the Thai FDA has long approved it as a traditional food ingredient. These regulatory developments underscore the transition of *W. globosa* from a regional delicacy to a globally recognized sustainable protein source. Nevertheless, the harmonization of labeling, nutrient claims, and health benefit substantiation remains critical to international trade. For nutraceutical applications, standardized compositional benchmarks and validated clinical evidence are required for any structure–function or therapeutic claim (Table 7).

## 8. Sustainability and Environmental Impact

**Figure 3:**
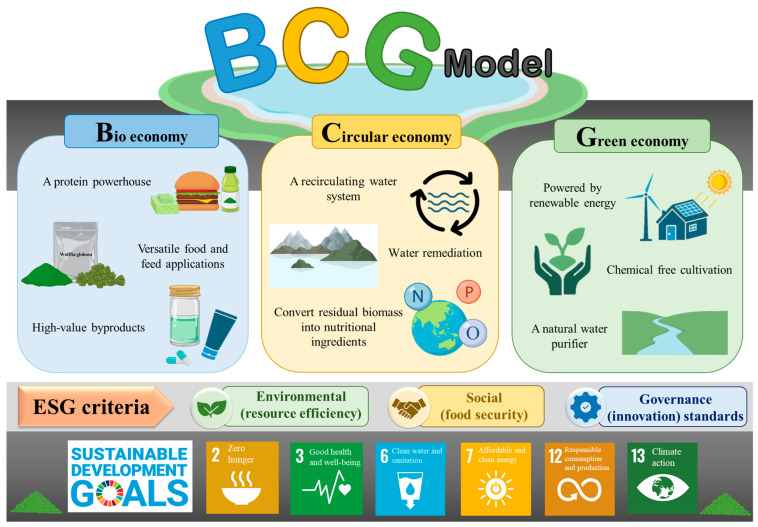
Biocircular-green economy (BCG) model for the sustainability and environmental impact of *W. globosa*. Adapted from Ujong et al. [8] Copyright 2025 Royal Society of Chemistry and the United Nations [76] Copyright 2025 United Nations.

*W. globosa* is a good alternative for creating a sustainable economy according to the Biocircular-Green Economy (BCG) approach, which can emphasize the efficient use of biological resources, reduce waste, and support the development of a green economy [67]. It can also be linked to Environmental, Social, and Governance (ESG) criteria (Figure 3). The use of recirculating water systems and renewable energy for *W. globosa* production can create jobs and economic opportunities for the community. The use of *W. globosa* in dietary supplements and meat substitutes provides nutritious food for people in areas with protein deficiency. Support for research and development of environmentally friendly watercress cultivation technologies, emphasizing quality control in production, and the use of innovation to increase production efficiency is needed. The use of environmentally friendly and traceable production methods ensures that industries adhere to ESG standards [68].

### 8.1. Bio-Economy

*W. globosa* is used in human food because of its high nutritional value and potential as a protein substitute for meat. *W. globosa* can be used as an ingredient in dietary supplements, such as protein powders for dietary supplements, or protein drinks for those seeking protein in their daily diets, such as athletes or the elderly who want to build muscle and improve their health [1,9]. *W. globosa* can be used to produce meat substitutes, or proteins with properties similar to meat, providing options for consumers seeking environmentally and animal-friendly alternatives [2]. *W. globosa* is also used in animal feeds to supplement protein, reducing reliance on plant and animal proteins that have environmental impacts [5]. The development of innovative production technologies can increase *W. globosa* production efficiency and maximize the use of biological resources. The development of *W. globosa* technology in systems with temperature, humidity, and light control allows for maximum production efficiency in limited spaces, such as hydroponics or aquaponics, which do not require large areas for cultivation. The use of advanced technologies, such as cold press or membrane filtration, to extract proteins and nutrients from water hyacinths can fully preserve their nutritional values. Promoting awareness of the benefits of *W. globosa* in the production of animal feed and supplements will help increase the popularity of *W. globosa* products in the market [5,65].

### 8.2. Circular Economy

Water recycling is an important aspect of a circular economy. *W. globosa* can utilize a recirculating water system to increase water efficiency. A recirculating water system on *W. globosa* farms filters and treats farm wastewater for reuse in subsequent cycles [4]. An aquaponics system, a recirculating water system used to grow both crops and fish, uses energy from the filtered water from fish farms [69]. This water is of high quality and can be reused multiple times. *W. globosa* has advantages in both its role as a food plant and as a water remediator in the circular bioeconomy and water restoration. *W. globosa* is an aquatic plant capable of producing highly nutritious biomass, such as protein and essential amino acids, which could be used as animal feed or even human food in the future, with significant potential for use in the circular economy. *W. globosa* can absorb toxins and excess nutrients from water, thus playing a crucial role in wastewater treatment. This is because aquatic plants absorb contaminants, such as nitrates and phosphates, which can contribute to water pollution. Growing *W. globosa* in polyethylene ponds reduces the accumulation of these toxins and improves water quality [70]. It can also be used in water recycling processes, making it ideal for farming in areas with limited water or production systems that require efficient water treatment [5]. The ability of plants to absorb excess nitrogen and phosphorus makes them ideal for integrated aquaculture systems and wastewater nutrient remediation [70]. *W. globosa* is a plant that thrives in the presence of nitrogen, phosphorus and oxygen. *W. globosa* has high efficiency in wastewater removal, with COD, total phosphorus (TP), and total nitrogen (TN) removal exceeding 80%, 90%, and 50%, respectively [8]. By converting residual nutrients into edible biomass, this process supports the principles of a circular economy, creating a closed nutrient cycle and reducing pollution levels. Biomass and crop residues can also be used as substrates. The production of biogas or organic fertilizer increases the sustainability of the system. Research on co-culture with microalgae or duckweed highlights the potential of multi-plant systems to optimize space and resources [5,8,70].

### 8.3. Green Economy

Solar panels can be used to generate electricity for water management systems on *W. globosa* farms, such as pumping, water circulation, or environmental control (e.g., temperature and humidity control), which will reduce fossil fuel electricity consumption and carbon dioxide emissions. Installing wind turbines in windy areas on *W. globosa* farms will provide a renewable energy source for electricity generation and environmental management in the region. Growing and caring for *W. globosa* in an environmentally friendly system without the use of chemicals helps to maintain environmental balance [71]. In general, the cultivation of crops, insects, and microorganisms imposes a lower environmental burden than livestock production [72]. Floating plants in the Lemnaceae family are effective at removing atmospheric CO_2_, treating wastewater, providing a source of raw materials for biofuel production, and providing superior nutritional quality for both humans and livestock. Floating plants in the Lemnaceae family require minimal space, light, and fertilizer. Furthermore, other environmental factors, such as nutrient availability, light, and microbiome presence, influence the response of *W. globosa* to elevated CO_2_ under conditions of high CO_2_, low nutrient availability, and moderate light intensities. The *W. globosa* microbiome maintains CO_2_ retention and relative growth rate as the incident light intensity increases (in the presence of high CO_2_ concentrations). The microbiome reduces the negative feedback on photosynthesis due to increased sugar accumulation in plants. *W. globosa* also exhibits a clear tendency to uptake ammonium over nitrate [73]. Water hyacinth cultivation can be further optimized using hydroponic vertical farms, where nutrients and water are recycled, saving resources, space, and energy for the production of high value products [73]. Traditional animal protein sources have farming processes that inevitably have environmental impacts, including high greenhouse gas emissions, large water consumption, and land requirements [10]. *W. globosa* is a high-quality, environmentally friendly food crop that can produce higher protein than soybeans when grown on the same area. The cultivation of *W. globosa* requires less water and land than other protein crops, thus conserving the agricultural land. The estimated yields are 20–50 L/kg [74] compared to more than 2000 L/kg for soybeans [75] and over 15,000 L/kg for beef [10]. *W. globosa*’s rapid growth and high nitrogen uptake efficiency reduce fertilizer use and nutrient losses, while closed-loop systems can reduce greenhouse gas emissions to near zero [74]. Life cycle assessment (LCA) of *W. globosa* cultivation produces fewer greenhouse gas emissions than animal protein and less than soy [2]. Therefore, the commercial production of *W. globosa* aligns with the United Nations Sustainable Development Goals (SDGs), including SDG 2 (Zero Hunger), SDG 3 (Good Health and Well-being), SDG 6 (Clean Water and Sanitation), SDG 7 (Affordable and Clean Energy), SDG 12 (Responsible Consumption and Production), and SDG 13 (Climate Action) [8,76].

## 9. Challenges, Gaps, and Future Perspectives

The major challenges in facilitating the progress of *W. globosa* as a key ingredient for commercial use are still limited due to the absence of standardized compositional information. Furthermore, the variation in the chemical composition, particularly protein, amino acids, and phytochemicals in *W. globosa*, strongly influences this challenge, and it is diversified based on water quality, nutrient availability, temperature, and light. This causes difficulties in the quality verification and standardization of nutrient labeling for commercial purposes. Therefore, studies should focus on the standardization of *W. globosa* growing practices and reliable methodological analyses to establish reliable reference values. Furthermore, identifying the metabolomic and proteomic profiles of *W. globosa* under various environmental conditions could help understand how abiotic stressors induce or affect secondary metabolism and the synthesis of bioactive compounds in *W. globosa*. This information could be helpful and lead to the enhancement of desired nutrients in targeted food production. Although several studies have shown that *W. globosa* exhibits strong antioxidant and antihypertensive properties and regulates the glycemic index in vitro and in animal studies, human clinical evidence remains limited. Furthermore, current studies have limited sample sizes and durations and often focus on short-term markers of health, mainly postprandial glycemia or satiety. There is a clear need for RCTs to confirm health claims and to assess long-term physiological benefits. Mechanistic studies should also examine peptide bioavailability, metabolite turnover, and molecular targets involved in metabolic regulation (e.g., Nrf2, AMPK, and gut–brain axis targets). It is important to establish dose–response relationships and safety buffers to support the labeling of functional or nutraceutical products by the EFSA and the FDA. Despite its positive nutrient profile, processing remains a challenge. The small size and high moisture content of Wolffia makes drying high-energy, and sufficient chlorophyll can add a green color and grassy flavor to food products. Advanced low-temperature drying, enzyme-assisted dechlorophyllization, and flavor-masking technologies (e.g., microencapsulation or complexation with starches and cyclodextrins) show promise for developing improved sensory qualities of green tea. However, there has been limited research on protein extraction and texturization. However, the development of scalable methods for producing isolates and textured protein analogs with improved functionality (i.e., solubility, gelling, and emulsification) will facilitate their incorporation into plant-based meat and dairy alternatives.

*W. globosa* has been approved and recognized by the EFSA and GRAS, although its regulatory agreement across regions has not yet been fully achieved. The commercial products and their approved nutritional or medicinal uses have not yet been determined. Furthermore, official monographs for *W. globosa* products are not fully available in food composition databases such as the United States Department of Agriculture (USDA), FAO, and the International Network of Food Data Systems (INFOODS), limiting broader industry adaptations. Therefore, it is essential to bring academia, regulatory agencies, and private sectors together to create worldwide safety and compositional guidelines for *W. globosa* to increase consumer confidence in its products, which still exhibits a clear gap in consumer perception research. Additionally, there is little to no marker or consumer familiarity with *W. globosa* products beyond Asia, even though they are considered and presented to consumers as green caviar. To improve the market penetration of *W. globosa*, effective communication strategies that highlight sustainability, nutrient density, and natural resources are required. Currently, the cost of *W. globosa*-based food products remains high due to the lack of specialized infrastructure, the requirement for controlled environment systems, and the need for human labor for harvesting. Scaling up production from pilot facilities to industry-wide commercialization will require the mechanization of harvesting, dewatering, and drying. It is essential to conduct economic modeling and techno-economic assessments comparing the *W. globosa* supply chain with existing plant protein supply chains to demonstrate their competitiveness. Investment in biorefinery concepts, where Wolffia biomass is fractionated into proteins, fibers, pigments, and bioactives, has the potential to simultaneously increase the profitability and sustainability of the industry.

## 10. Conclusions

*W. globosa* represents one of the most promising and sustainable plant-based protein sources for the future of human nutrition. Its unparalleled productivity, high-quality protein composition, rich micronutrient content, and bioactive compounds make it a viable alternative to traditional legume- and animal-derived proteins. Beyond its nutritional value, Wolffia offers significant environmental advantages, including minimal water and land use, rapid growth, and potential integration into circular bioeconomy systems. Evidence to date supports its antioxidant, anti-inflammatory, antihypertensive, and glycemic-regulating activities, validating its dual role as a functional food ingredient and nutraceutical resource. Regulatory recognition by the EFSA and GRAS designation in other jurisdictions further underscores its safety and readiness for commercial development. However, large-scale implementation will require addressing knowledge gaps in compositional standardization, sensory optimization, and clinical substantiation of health benefits. The convergence of sustainability, nutrition, and innovation positions *W. globosa* as a symbol of next-generation food systems. Continued collaboration among researchers, industries, and policymakers will be critical to unlocking their full potential. As the global population demands eco-efficient protein sources, *W. globosa*, the “green caviar”, stands poised to redefine the intersection of food, health, and environment. If produced under controlled conditions and supported by standardized specifications and human substantiation, *W. globosa* could contribute to diversified and sustainable protein supply through incorporation into foods where high-quality protein and low-allergen positioning are desirable.

## Figures and Tables

**Table 1:** Cultivation systems of *W. globosa*.

Cultivation Systems	Natural Water Bodies	Open-Field Cultivation Systems [16]	Hydroponic Greenhouse Cultivation [22]	Indoor Vertical Farm (IVF) [19]	Bioreactor System [20,21]
System description	Stagnant freshwater bodies (ponds, natural wetlands)	Cultivation in polyethylene tanks with fertilizer application	Cultivation under fully or semi-controlled greenhouse conditions	Closed system using artificial lighting and nutrient solutions	Cultivation in controlled bioreactors
Production system	Outdoor, open system	Outdoor, open system	Indoor, fully or semi-controlled system	Indoor, closed system	Indoor, closed system
Level of production factor control	Limited	Moderate-High	Moderate-High	High	Very high
Fertilizer and environmental management	Uncontrolled	Basic fertilizer management (e.g., NPK)	Controlled fertilization and partial environmental control	Precise nutrient and environmental management	Strict control of nutrients and environmental conditions
Yield	Variable	High	High	High	High and high quality
Nutritional value and bioactive compounds	Inconsistent nutritional value	High protein content and amino acids	Relatively stable nutritional quality	Adjustable nutritional and bioactive profiles	Targeted bioactive compound production
Advantages	Low cost, traditional practice	Simple system, low investment, high biomass	Controlled environment; pest and disease protection	High safety, year-round production	High purity, suitable for high-value products
Limitations	High contamination risk, unstable supply	Low level of control over production factors (e.g., fertilizers, light, rainfall, water quality)	Higher cost than outdoor systems	High capital and energy costs, Requires skilled operators	High operational cost; contamination risk; requires multidisciplinary expertise
Suitability for application	Household consumption	Human food, animal feed	Commercial food production	High-quality food, industrial production	Pharmaceutical and biotechnological applications

**Table 2:** Proximate, amino acid composition, phytosterol, phenolic acids, and flavonoid content of *W. globosa* [7].

Proximate Composition	g/100 g DW	Proximate Composition	g/100 g DW
Moisture	3.73–4.25	Ash	6.76–7.84
Protein	20.55–22.74	Fiber	15.76–16.53
Lipid	3.23–4.08	Carbohydrate	50.73–52.44
**Essential amino acids**	**g/100 g DW**	**Non-Essential amino acids**	**g/100 g DW**
Arginine	1.32–2.13	Alanine	1.07–2.05
Histidine	0.24–0.42	Asparagine	0.36–0.99
Isoleucine	0.92–1.32	Aspartic acid	0.51–0.75
Leucine	0.871.24	Cysteine	0.03–0.07
Lysine	0.29–0.47	Glutamine	0.62–1.08
Methionine	0.14–0.18	Glutamic acid	0.49–0.77
Phenylalanine	1.01–1.36	Glycine	0.17–0.27
Threonine	0.12–0.22	Proline	0.03–1.00
Tryptophan	0.45–0.58	Serine	0.19–0.37
Valine	1.03–1.67	Tyrosine	1.45–1.66
**Phenolic acid content**	**µg/g DW**	**Phenolic acid content**	**µg/g DW**
Gallic acid	169.86–173.20	Vanillin	ND
Protocatechuic acid	166.94–172.49	p-Coumaric acid	55.36–59.80
p-Hydroxybenzoic acid	ND	Ferulic acid	18.02–22.63
Vanillic acid	78.90–86.20	Sinapic acid	129.26–137.05
Caffeic acid	12.62–13.69	Cinnamic acid	57.04–61.38
Syringic acid	9.61–10.71	Gentisic acid	20.72–26.64
**Flavonoid content**	**µg/g DW**	**Phytosterols**	**µg/100 g DW**
Rutin	81.30–89.73	Campesterol	ND-212.45
Catechin	ND	Stigmasterol	ND-212.90
Quercetin	176.77–195.36	β-Sitosterol	101.96–625.08
Apigenin	172.55–179.98	Cycloartenol	ND-43.74
Kaempferol	38.40–42.46	Brassicasterol	ND-29.88
Total	473.08–503.79	ND: not determined	

**Table 3:** Functional Properties of *W. globosa* Compared with Soy, Pea, and Spirulina.

Functional Category	*W. globosa* [7,8,9,10,11,12,13,14,15,16,17,18,19,20,21,22,23,24,25,26,27,28,29,30,31,32,33,34,35,36,37,38,39]	Soy [40]	Pea [41]	Spirulina [9]	Reference
Protein content	20.55–31.50 g/100 g DW	≈40% DB (whole seeds); ≈90% (protein isolate)	20–30% DB (whole seeds); 81–89% (protein isolate)	50–70% DB (≈65.3 g/100 g powder)	[7,27,40,41]
Amino acid profile/protein quality	Rich in arginine, valine, and leucine; PDCAAS ≈ 0.89	Complete essential amino acid profile; PDCAAS ≈ 0.92–1.00	Dominated by legumin (11S) and vicilin (7S) storage proteins	Rich in phycobiliproteins, particularly C-phycocyanin	[9,27,39,41]
Lipid composition	3.23–5.18 g/100 g DW; high α-linolenic acid (C18:3 ω-3)	≈0.8 g/100 g (isolate); residual plant oil	5.1–55.0 g/kg (cultivar- and process-dependent isolates)	≈0.8 g/100 g DW; enriched in ω-3 fatty acids	[7,40,41]
Carbohydrates	50.73–52.59 g/100 g DW	Very low in isolate form due to removal during processing	4–39 g/kg wb (isolate)	≈12.8 g/100 g DW	[9,27,39,40]
Dietary fiber	10.7–16.5 g/100 g DW; predominantly insoluble fiber	~0 g/100 g in protein isolate	Low or trace levels in isolate form	Minor amounts; not nutritionally significant	[9,27,39,40]
Micronutrients	Vitamin B_12_, β-carotene, α-tocopherol; Fe, Ca, Mg, Zn	Ca (~178 mg), Fe (~14.5 mg), Mg (~39 mg), P (~776 mg)/100 g isolate; vitamin B_12_ absent	Trace minerals depending on processing; generally reduced in isolate form	Vitamin B_12_, vitamin A, Fe, Mg	[27,39,40,41,42]
Phytochemicals	Phenolics (gallic acid), flavonoids (rutin, quercetin, apigenin), phytosterols	Isoflavones largely removed in isolate form	Phenolics largely reduced during protein isolation	Chlorophyll, C-phycocyanin	[27,40,41,42]
Functional properties	Enzymatic hydrolysis improves solubility and emulsifying activity	EAI ≈ 38.6 m^2^/g; ESI ≈ 19.5 min (isolate)	EAI ≈ 31–39 m^2^/g; ESI ≈ 11 min (cultivar-dependent)	High-pressure homogenization (50 MPa) markedly improves EAI, oil-holding, and foaming capacity	[27,39,40,41]

DW: dry weight; DB: dry basis; wb: wet basis; PDCAAS: Protein Digestibility-Corrected Amino Acid Score; EAI, emulsifying activity index; ESI, emulsifying stability index. Nutrient composition may vary depending on the cultivar, growth conditions, and protein extraction or processing method.

**Table 4:** Identified bioactive compounds and fractions of *W. globosa* and their functional and biological activities.

Bioactive Compounds Identified	Functional and Biological Activities	Extraction/Modification Method	Microbial or Cell Line Target	References
Protein concentrate hydrolysate (PCH), protein extract (PE), and protein solution (PS) rich in aspartic acid and glutamic acid; peptides	Antimicrobial activity, antioxidant activity, and functional properties (high solubility, emulsifying capacity, and stability)	Ultrasound-assisted extraction (UAE), isoelectric precipitation, and Alcalase hydrolysis	Vibrio parahaemolyticus, Candida albicans, and other foodborne pathogens	[33]
Indole-3-acryloylglycine (I3AG), spiculosine (ES-285), and selenium-enriched phenolics	Antioxidant and anti-proliferative effects	Ultrasonic-assisted extraction (UAE) with 60% methanol	Human ovarian cancer cells (A2780 and SKOV3)	[45]
Essential amino acids (leucine, valine, phenylalanine)	Antioxidant, foaming, and emulsifying activities; anti-cancer potential	Ultrasonic-assisted extraction (UAE) and enzymatic hydrolysis (Alcalase and Protamex)	Human ovarian cancer cell lines (A2780)	[1]
Bioactive vitamin B12 (hydroxocobalamin, 5-deoxyadenosylcobalamin, methylcobalamin, cyanocobalamin)	Bioavailable B12 source (increases serum B12 levels in humans)	Cultivation in controlled greenhouse systems	Humans (clinical trial participants)	[50]
Protein extracts (precipitates and supernatants) and phenolic compounds	Antioxidant activities (ABTS/FRAP) and anti-inflammatory effects (reduced IL-1β and IL-6)	Alkaline extraction followed by acid (pH 3/5) or heat (85 °C) precipitation	THP-1 human monocytic cell line	[46]
72 phenolic compounds (18 phenolic acids, 29 flavonoids, 25 polyphenols), carotenoids, and vitamin C	Antioxidant activity (DPPH, ABTS, FRAP)	Boiling, Freeze-thawing, and Mechanical crushing	Not in source	[43]
Essential amino acids (leucine, lysine, valine), dietary fiber, and phenolics	Antioxidant activity (ORAC/FRAP) and nutritional fortification	Freeze-dried powder (WP) in snack formulation	*S. typhimurium* (TA98, TA100, TA102, TA1535, TA1537) for Ames test	[27]
Phenolic acids (protocatechuic, vanillic, p-coumaric), rutin, and alpha-linolenic acid	Antioxidant activity (DPPH and FRAP assays)	Hot air oven drying at 60 °C	Not in source	[7]
Alpha-tocopherol, gamma-oryzanol, and vicilin-like protein	Antioxidant capacity, water/oil holding capacity, and emulsifying abilities	Freeze-drying	Not in source	[44]
Essential amino acids and Vitamin B12	Protein bioavailability and amino acid intake	Not in source	Human subjects (randomized controlled trial)	[31]
Beta-sitosterol and stigmasterol	Anti-inflammatory activity (nitric oxide inhibition)	Not in source	Macrophage cells (RAW 264.7) and Human dermal fibroblast (HDFn)	[6]

**Table 6:** Impact of processing on nutritional and functional qualities.

Processing Method	Condition	Nutritional and Functional Quality	References
Drying	50 °C for 6 h	Total phenolics, flavonoids, and chlorophylls contents of 55.28 ± 1.35 (μg gallic acid equivalent (GAE)/g dry weight), 159.84 ± 6.65 (μg catechin equivalent (QE)/g dry weight) and 22.91 ± 0.15 (mg/g dry weight), respectively.	[59]
Pre-treated with salt solution combined with blanching	Pre-treated with blanching (80 °C, 3 min) in the different salt solutions before drying in a hot air oven at 60 °C for 8 h	The number of antioxidants from DPPH and FRAP method were 11.64 and 22.55 mmol Trolox eq/kg, respectively and TPC was 454.26 mg GAE/100 g.	[57]
Boiling	100 °C for 10 min before drying at 50 °C for 12 h.	The highest and most prominent leaching of crude lipid, mineral (ash), DPPH and FRAP antioxidant activities, TPC, total tannin content, and TFC.	[43]
Freeze–thawing	−20 °C for 12 h; thawing in tap water at room temperature for 2–3 h before being placed at 50 °C for 12 h.	The highest contents of crude protein, crude lipid, total carotenoids, vitamin C, Chl-*a*, and various phenolic compounds, flavonoids, and other polyphenols.	[43]
Mechanical crushing	Grounded/blended into batches of 500 g before during at 50 °C for 12 h.	The lowest leaching of TPC, TFC, and total tannin content but the highest leaching of the crude protein, total carotenoids, Chl-*a*, and Chl-*b*.	[43]
Drum drying	120 °C–150 °C	Proteomic analysis identified 342 proteins, including ribulose-1,5-bisphosphate carboxylase oxygenase (RuBisCO), adenosine triphosphate (ATP) synthase, and antioxidant enzymes, reflecting its photosynthetic activity and functional potential. All essential amino acids were retained in dried samples, and the antioxidant activities increased after post-drying.	[60]
Ultrasound-assisted extraction (UAE)	At 120 kHz (power level 4) conducted at room temperature for 15 min before isoelectric precipitation (pH 3.5)	The highest protein solubility relative to the protein extract, total amino acids in the protein concentrate hydrolysate increased, whereas the protein solution contained lower totals of amino acids.	[33]
Ultrasound-assisted extraction	Solid–liquid ratio of 0.1:10 g/mL, ultrasound amplitudes of 100% and extraction time of 30 min	The maximum protein yield was found to be 39.65% *w*/*w*. The major amino acids were glutamate, aspartate and leucine.	[61]
High-pressure processing (HPP)	At 450 MPa for 5 min	Enhanced water holding capacity (WHC), oil holding capacity (OHC), and protein digestibility (up to 83.83%). Lowered phytic and oxalic acids, improving mineral bioavailability.	[11]
Steaming	At 100 °C for 10 min	Improved EAA content, steaming best preserved chlorophyll (2.02 ± 0.10 mg/g DW) and reduces magnesium (Mg^2+^) loss.	[11]
Boiling	At 100 °C for 10 min	Led to structural damage, loss of bioactives, and mineral leaching, reducing the overall nutritional value.	[11]
Tray drying	At 70 °C for 5 h		[11]

**Table 7:** Safety and toxicological data of *Wolffia* spp.

Assessment Category	Reported Findings	References
Heavy Metal Contamination	Accumulation risk: Duckweeds (*Wolffia* spp.) demonstrate high potential for the uptake and accumulation of heavy metals and organic pollutants, particularly Manganese (Mn). Water quality control during cultivation is critical to ensure final product safety.	[13,62]
Microbiological Safety	Process control required: Cultivation and processing must be strictly controlled to mitigate risks associated with microbial contamination and the potential presence of toxins such as microcystins.	[62]
Anti-nutritional Factors	Low levels & mitigation: Anti-nutritional factors (e.g., phytic acid, tannins, oxalates) are present at low concentrations. Processing techniques, such as specific extraction or fermentation, are effective in further reducing their content.	[52]
Allergenicity	Allergenic potential: The EFSA Panel noted a hypothetical concern that the high protein content might trigger allergic reactions (consistent with Novel Food principles for new protein sources) and recommended monitoring post-market.	[62]
Animal Toxicology	No adverse effects: A 90-day repeated-dose oral toxicity study in rats (GLP-compliant) established a No-Observed-Adverse-Effect-Level (NOAEL) at the highest tested dietary inclusion (20% *w*/*w*), demonstrating subchronic safety.	[35]
Genotoxicity Testing	Negative/non-genotoxic: Both in vitro (Ames test and micronucleus assay) using the primary Mankai product and screening of a finished snack formulation yielded negative results, indicating no genotoxic potential.	[27,35]
Human Safety Trials	Good tolerance & bioavailability: Short-term human randomized controlled trials (RCTs) reported good tolerance with no significant gastrointestinal adverse events, confirming the protein is bioavailable. Metabolic benefit: Short-term RCTs reported a metabolic benefit, specifically lower postprandial glucose peaks, without safety concerns during the trial period.	[16,36]
Regulatory Status	Approved as a novel food by the European Union (Regulation EU 2022/2223) and recognized as GRAS in the United States. In Thailand, it has long been traditionally consumed.	[62,66]

## Data Availability

No new data were created or analyzed in this study.

## Abbreviations

ABTS	2,2′-Azino-bis(3-ethylbenzothiazoline-6-sulfonic acid
ACE	Angiotensin-converting enzyme
AN	Ammoniacal nitrogen
BCG	Biocircular-green economy
BOD	Biological oxygen demand
BPA	Bisphenol A
BPH	Benzophenone
Ca	Calcium
Cd	Cadmium
COD	Chemical oxygen demand
Cr	Chromium
Cu	Copper
DEET	N, N-Diethyl-m-toluamide
DPPH	2,2-Diphenyl-1-picrylhydrazyl
DW	Dry weight
EAAs	Essential amino acids
EAI	Emulsifying activity index
EC_50_	Half maximal effective concentration
EFSA	European food safety authority
END	Endosulfan
ESG	Environmental, Social, and Governance Criteria
FDA	The Food and Drug Administration
Fe	Iron
FRAP	Ferric reducing ability power assays
GAE	Gallic acid equivalent
GAP	Good Aquaculture Practices
GRAS	Generally Recognized As Safe
HACCP	Hazard analysis and critical control point
I	Iodine
Ile–Pro–Pro	Isoleucine–proline–proline
INFOODS	International Network of Food Data Systems
K	Potassium
LCA	Life Cycle Assessment
LDL	Low-density lipoprotein
Mg	Magnesium
Mn	Manganese
N	Nitrogen
Na	Sodium
ND	Not determined
NEAA	Non-essential amino acids
NF-κB	Nuclear Factor kappa-light-chain-enhancer of activated B cells
NOAEL	No observed adverse effect level
Nrf2–ARE	Nuclear factor erythroid 2-related factor 2 and Antioxidant Response Element
OHC	Oil holding capacity
OMPs	Organic micropollutants
P	Phosphorus
PDCAAS	The Protein Digestibility-Corrected Amino Acid Score
PE	Polyethylene
PPO	Polyphenol peroxidase
PUFAs	Polyunsaturated fatty acids
QE	Catechin equivalent
RCTs	Randomized controlled trials
RuBisCO	Ribulose-1,5-bisphosphate carboxylase oxygenase
SCFAs	Short-chain fatty acids
SDGs	The United Nations Sustainable Development Goals
Se	Selenium
SELENBP1	Selenium-binding protein 1
TFC	Total flavonoid content
TN	Total nitrogen
TP	Total phosphorus
TPC	Total phenolic content
TRC	Triclosan
TSS	Total suspended solids
USDA	United States Department of Agriculture
Val–Pro–Pro	Valine–proline–proline
WHC	Water holding capacity
Zn	Zinc
